# More expressions of BDNF and TrkB in multiple hepatocellular carcinoma and anti-BDNF or K252a induced apoptosis, supressed invasion of HepG2 and HCCLM3 cells

**DOI:** 10.1186/1756-9966-30-97

**Published:** 2011-10-14

**Authors:** Dawei Guo, Xuezhong Hou, Hongbin Zhang, Wenyu Sun, Lei Zhu, Jian Liang, Xiaofeng Jiang

**Affiliations:** 1 Department of General Surgery, the Fourth Affiliated Hospital of China Medical University, Shenyang, China; 2 Department of General Surgery, the Chinese People's Liberation Army 463th hospital, Shenyang, China

## Abstract

**Background:**

Brain-derived neurotrophic factor (BDNF) and its receptor Tropomysin-related kinase B (TrkB) are commonly up-regulated in a variety of human tumors. However, the roles of BDNF/TrkB in hepatocellular carcinoma (HCC) have been poorly investigated.

**Methods:**

We evaluated the expressions of BDNF and TrkB in 65 cases of HCC by immunohistochemical staining. Moreover, in human HCC cell lines of HepG2 and high metastatic HCCLM3, the secretory BDNF in supernatant was measured by ELISA, the effects of BDNF neutralizing antibody or Trk tyrosine kinase inhibitor K252a on apoptosis and invasion were examined by flow cytometry and transwell assay respectively.

**Results:**

Higher expression of BDNF (63.1%) or positive expression of TrkB (55.4%) was found in HCC specimens, which was significantly correlated with multiple and advanced stage of HCC. BDNF secretory level in HCCLM3 was higher than that in HepG2 cells. Both anti-BDNF and K252a effectively induced apoptosis and suppressed invasion of HepG2 and HCCLM3 cells.

**Conclusions:**

These findings suggested that BDNF/TrkB are essential for HCC cells survival and invasion. BDNF/TrkB signaling should probably be an effective target to prevent HCC advancement.

## Background

Hepatocellular carcinoma (HCC) is a leading cause of cancer death worldwide, and the presense of intraheptatic metastases at the time of surgery has been regarded as the main causes of recurrence [1]. The cancer cells readily disseminate via portal venous branches and patients with multiple tumor nodules in liver are proved to have poor prognosis [2]. Multiple hepatocellular carcinoma is usually regarded as HCC with multiple tumor nodules, clinically classified as either intrahepatic metastasis or multicentric carcinogenesis [3]. Tumor cells' invasion into blood vessels and survival inside are essential to a successful metastasis in liver, resulting in the formation of intrahepatic metastases [4]. However, the key points have not been well elucidated, and the investigation of mechanisms for multiple HCC may improve the prognosis of this severe disease.

Brain-derived neurotrophic factor (BDNF) is a member of nerve growth factor family, playing an important role in supporting survival and growth of neurons. Tropomysin-related kinase B (TrkB) is the primary receptor of BDNF, which functions as a tyrosine kinase. BDNF and TrkB are up-regulated in a variety of primary human tumors, including neuroblastoma [5], breast [6], bladder [7] and ovarian [8] cancers. In gastric cancer, a high level of TrkB expression was predicted for distant metastases and poor prognosis [9]. TrkB overexpression was also found in highly metastatic pancreatic cancer cells, which was presumed to mediate the clinical features of aggressive growth and metastasis of pancreatic cancer [10]. When activated by BDNF, TrkB induces the activation of downstream signaling molecules, such as Akt [11,12] and ERK [13,14], which elicits the differential regulation of various cellular activities, like cell proliferation [15], differentiation [16], apoptosis [17], and invasion [18]. TrkB signaling promotes cell survival in an anchorage-independent manner [19]. In HCC, the expressions of BDNF and TrkB were found up-regulated in detached HCC BEL7402 cell aggregations, which were able to resistant to detachment-induced apoptosis [20].

Despite the increasing evidence of BDNF and TrkB on tumor progression, whether they are involved in multiple HCC has not yet been determined. In the present study, the expressions of BDNF and TrkB in HCC specimens were examined, and by neutralizing BDNF or inhibiting TrkB kinase activity in HCC cell lines to observe the effects of BDNF/TrkB interruption on cell apoptosis and invasion.

## Methods

### HCC samples

A total of 65 HCC patients who had therapeutic resection from January 2006 to January 2011 were enrolled in this study. This study was approved by the Medical Research Ethics Committee of China Medical University and the informed consent was obtained from all patients. All of the enrolled patients underwent curative surgical resection without having chemotherapy or radiation therapy. Formalin-fixed paraffin-embedded sections of tumor were stained routinely with hematoxylin and eosin (HE), and reviewed by two senior pathologists in order to determine the histological characteristics and tumor stage according to the AJCC/UICC TNM staging system (2003, Edit 6). Clinicopathological information including tumor distribution (solitary or multiple nodules), differentiation, stage and lymph node metastasis was obtained from patient records, and listed in additional file 1.

### Immunohistochemistry

65 paraffin sections of HCC were deparaffinized and rehydrated routinely. The sections were incubated overnight at 4°C with primary rabbit polyclonal antibody detecting BDNF (1:100) or TrkB (1:50, both from Santa Cruz, USA), following 3% H_2_O_2 _and 5% goat serum treatment at 37°C for 30 min after antigen recovery. Then they were incubated with second antibody and streptavidin-peroxidase (SP) complex for 30 min (SP kit, Maixin, China), and visualized with 3,3'-diaminobenzidine (DAB, Maixin, China). All the immunoreactions were separately evaluated by two senior pathologists. Cells with brown particles appearing in cytoplasm or cell membrane were regarded as positive. The intensity of BDNF immunostaining (1 = weak, 2 = intense) and the percentage of positive tumor cells (0-5% = 0, 6-50% = 1, ≥51% = 2) were assessed in at least 5 high power fields (×400 magnification) [7]. The scores of each tumorous sample were multiplied to give a final score of 0, 1, 2, or 4, and the tumors were finally determined as negative: score 0; lower expression: score ≤ 2; or higher expression: score 4. The percentage of TrkB positive tumor cells was assessed in at least 5 high power fields (×400 magnification), and >10% was regarded as positive sample [21].

### Cells culture and treatments

Human HCC cell lines HepG2 and HCCLM3 (with high metastatic potential) were purchased from KeyGen (China). HepG2 cells were grown in RPMI-1640 (Invitrogen, USA) and HCCLM3 cells were cultured in DMEM (high glucose, Invitrogen, USA) supplemented with 10% FBS, in incubator with 5% CO_2 _at 37°C. To neutralize secretory BDNF in culture supernatant for subsequent studies, cells (80-90% confluence) were treated with anti-BDNF antibody (20 μg/ml, Santa Cruz, USA) for 24 h. To interfere with receptor tyrosine kinase signaling, cells were also treated by Trk tyrosine receptor kinase inhibitor K252a (0.1 μM, Sigma, USA) for 24 h. Cells treated were used for apoptosis or invasion assays as described below. The examinations were repeated at least three times.

## Elisa

Human BDNF Quantikine™ ELISA kit purchased from R&D Systems was used in this study. HepG2 and HCCLM3 cells were cultured for 24 h before the supernatant was collected by centrifugation. BDNF secretion was measured using ELISA. In brief, 50 μl of samples or standard was added to the microplate wells with 100 μl assay diluent and incubated at room temperature for 2 h, and 100 μl of BDNF conjugate was added. Incubation was continued at room temperature for 1 h. Microplates were washed and developed using 200 μl of substrate solution. Then the optical density was read at 450 nm and wavelengh correction was set to 570 nm using a microplate reader.

### Cell apoptosis assay

The cell apoptosis was examined by flow cytometry using an Annexin V-FITC apoptosis detection kit (BD, USA), following the manufacturer's protocol. Cells were washed twice in ice-cold PBS and resuspended in 1 × binding buffer (1 × 10^6^/ml). Cells of 100 μl (1 × 10^5^) were gently mixed with 5 μl Annexin V-FITC and 5 μl PI, and then incubated for 15 min at room temperature away from light. After supplemented another 400 μl 1 × binding buffer, cell apoptosis was detected in flow cytometer. Data are representative of three individual experiments.

### Cell invasion assay

The cell invasion assay was performed using a 24-well Transwell chamber (Costar, USA). At 24 h following anti-BDNF treatment, cells (1 × 10^4^) were detached and seeded in the upper chamber of a 8 μm pore size insert precoated with Matrigel (BD, USA) and cultured in serum-free medium for 24 h. Cells were allowed to migrate towards medium containing 10% FBS in the bottom chamber. The non-migratory cells on the upper membrane surface were removed with a cotton tip, and the migratory cells attached to the lower membrane surface were fixed with 4% paraformaldehyde and stained with crystal violet. The number of migrated cells was counted in 5 randomly selected 200× power fields under microscope. Data presented are representative of three individual wells.

### Statistical analysis

The SPSS 13.0 software was applied to complete data processing. χ2-test was applied to analyze the correlations between BDNF or TrkB expression and clinicopathological characteristics. T-test was used to evaluate the difference of BDNF secretion between HepG2 and HCCLM3 cells. One-way ANOVA was used to compare the differences between cells with various treatments. All data were represented as mean ± SD and results were considered statistically significant when the p-value was less than 0.05.

## Results

### The expressions of BDNF and TrkB in 65 cases of HCC by immunohistochemistry

**Figure 1:**
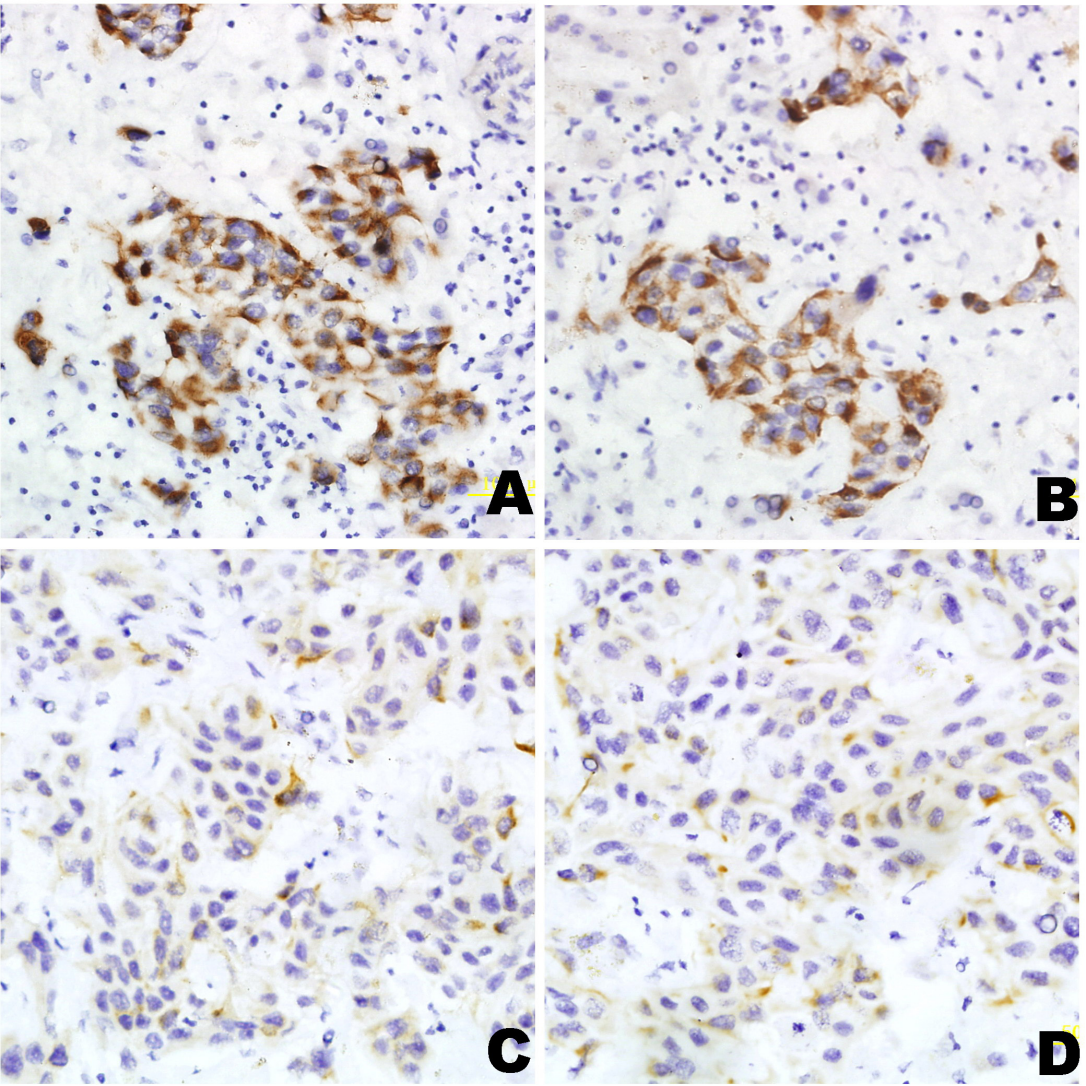
**BDNF and TrkB expressions in HCC by immunohistochemistry**. A and B, high BDNF and TrkB immunoreactivity in multiple HCC. C and D, positive BDNF and TrkB immunostaining in solitary HCC. Original magnification: all ×400.

BDNF was expressed in 57 (87.7%) HCC samples. We considered that 41 (63.1%) cases of HCC were higher expression (scores of 4) and 24 cases (36.9%) were lower expression (scores of 0, 1 or 2), including negative ones, as described in Materials and methods. The positive expression rate of TrkB in HCC tissues was 55.4% (36/65), and 44.6% were negative (26/65), as described in Materials and methods. Since BDNF/TrkB have been reported to facilitate survival and metastasis of tumor cells [22], the association between BDNF or TrkB expressions and the presence of intrahepatic dissemination at the time of resection was analyzed statistically in the present study. More cases of intrahepatic multiple tumors were found in HCCs with BDNF higher expression (p = 0.002). Likewise, HCCs with negative TrkB tended to be solitary tumors (p = 0.049). In addition, patients with more BDNF or positive TrkB expression had advanced stage of HCC (p = 0.005, p = 0.013). Moreover, a significant difference of BDNF, not TrkB expression was detected between variously differentiated HCCs (p = 0.036), and between HCCs with or without lymph node metastasis (p = 0.016). Samples of BDNF and TrkB expression in HCCs are shown in Figure 1. The correlations of BDNF or TrkB expression and clinicopathological characteristics are shown in Table 1 and 2.

**Table 1:** Clinicopathological characteristics and BDNF expression by immunohistochemistry in 65 cases of HCCs.

		BDNF		
		
		Higher expression (n = 41)	Lower expression (n = 24)	p-value
Distribution	Solitary	10	15	*0.002
	Multiple	31	9	
Differentiation	Well	23	7	*0.036
	Moderate-poor	18	17	
Stage	I+II	7	12	*0.005
	III	34	12	
Lymph node metastasis	+ -	19 22	4 20	*0.016

* = statistically significant difference.

**Table 2:** Clinicopathological characteristics and TrkB expression by immunohistochemistry in 65 cases of HCCs.

		TrkB		
		
		Positive expression (n = 36)	Negative expression (n = 29)	p-value
Distribution	Solitary	10	15	*0.049
	Multiple	26	14	
Differentiation	Well	20	10	0.090
	Moderate-poor	16	19	
Stage	I+II	6	13	*0.013
	III	30	16	
Lymph node metastasis	+ -	14 22	9 20	0.510

* = statistically significant difference.

### The secretion of BDNF in HepG2 and HCCLM3 cells by ELISA

BDNF is a cytokine secreted by a few human cancers, supporting growth and survival of tumor cells [23]. To explore whether HCC cells express BDNF secretorily, BDNF in the supernatant of HepG2 and HCCLM3 cells was examined by ELISA assays. The amounts of BDNF produced extracellularly by HepG2 and HCCLM3 cells were 88.6 ± 14.4 pg/ml and 138.4 ± 22.2 pg/ml, respectively (p = 0.031), which was shown in Table 3. This result showed that HCCLM3 cells had more BDNF production, which probably correlated with its high metastatic potential.

**Table 3:** Secretion of BDNF in supernatant of HepG2 and HCCLM3 cells by ELISA.

Cells	BDNF concentration (pg/ml)	p value
HepG2	88.6 ± 14.4	*0.031
HCCLM3	138.4 ± 22.2	

* = statistically significant difference.

### Anti-BDNF or K252a promoted cell apoptosis

**Figure 2:**
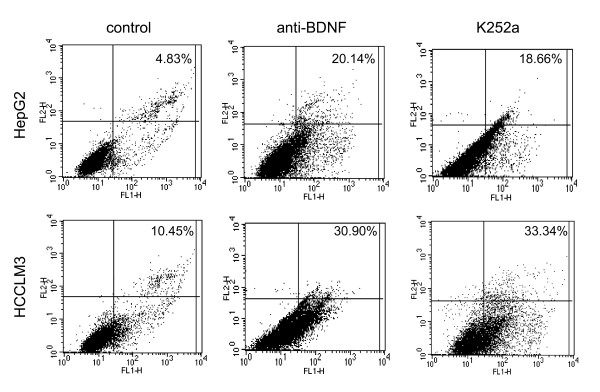
**Anti-BDNF or K252a treatment promoted cell apoptosis**. The apoptotic cells in anti-BDNF or K252a group were apparently increased in HepG2 or HCCLM3, in contrast to those control cells. The results were indicated as mean ± SD of three individual tests.

It was demonstrated BDNF/TrkB protected various tumor cells from apoptosis [24]. To investigate a positive role of BDNF/TrkB in HCC cell survival, apoptosis was examined after anti-BDNF or K252a treatment using Annexin V-FITC assay by flow cytometry. The apoptotic rates of control, anti-BDNF and K252a treated HepG2 at 24 h time point were 5.29 ± 0.54%, 20.21 ± 1.54%, 18.39 ± 0.83%, respectively (p = 0.000, Figure 2). And the apoptotic rates of control, anti-BDNF and K252a treated HCCLM3 at 24 h time point were 10.88 ± 0.42%, 30.35 ± 1.60%, 31.37 ± 2.16%, respectively (p = 0.000, Figure 2). These results suggested that neutralizing antibody specific for BDNF or Trk tyrosine kinase inhibitor K252a against TrkB probably antagonized the protection of BDNF/TrkB for HCC cells.

### Effect of anti-BDNF or K252a on cell invasion

**Figure 3:**
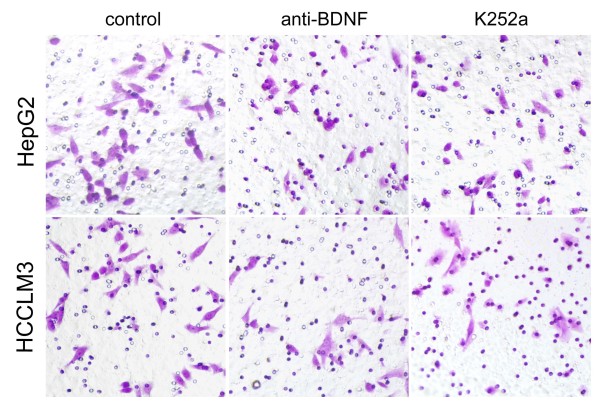
**Interruption of cell invasion by anti-BDNF or K252a treatment**. The number of invasive cells in anti-BDNF or K252a group was significantly reduced in HepG2 or HCCLM3, compared with that in control group. The values were mean ± SD of three replicates.

To understand the potential signaling induced by BDNF/TrkB that affects cell invasion, anti-BDNF or K252a was used and the invasion of treated cells was examined by Transwell assay. As shown in Figure 3, the invasive numbers of control, anti-BDNF and K252a treated HepG2 at 24 h were 42.3 ± 2.5, 30.7 ± 2.1 and 33.3 ± 1.5, respectively (P = 0.001). And the invasive numbers of control, anti-BDNF and K252a treated HCCLM3 cells at 24 h were 51.3 ± 3.2, 39.7 ± 1.5 and 42.7 ± 3.1, respectively (P = 0.005). These results showed that both anti-BDNF and K252a effectively interrupted the invasion of HepG2 and HCCLM3 cells.

## Discussion

Hepatocellular carcinoma is the most lethal malignancy in many countries, and the incurable feature is mainly due to the advanced stage of disease with metastasis at presentation. The intrahepatic dissemination of tumor cells is common in HCC patients with poor prognosis. It is rather necessary to clearly elucidate the molecular mechanisms that promoted HCC metastasis. BDNF and its high-affinity receptor TrkB are well studied to facilitate apoptosis resistance and metastatic tumor cells survival [25]. Aiming at interfering BDNF/TrkB signaling may be helpful in the progression of effective anticancer strategies [26,27].

In the present study, the expressions of BDNF and TrkB were examined in 65 cases of HCC by means of immunohistochemistry to evaluate the involvement of BDNF/TrkB in the progression of HCC. BDNF was found up-regulated and TrkB was overexpressed in human malignancies [21,28]. Our results showed that the expressions of both BDNF and TrkB appeared higher in multiple HCCs than those solitary tumors. A statistical difference in BDNF immunoreactivity not TrkB was observed between well and moderate-poorly differentiated HCCs. We also found a significant correlation between higher BDNF expression and lymph node metastasis. However, TrkB positive expression was not found difference in HCCs with lymph node metastasis or not. Moreover, BDNF and TrkB expressions were correlated with clinicopathological stage, and higher expressions of them in advanced HCCs were detected. These findings suggested a potential role of BDNF and TrkB in affecting intrahepatic dissemination of HCC cells.

Then HepG2 and HCCLM3 cells were utilized to assess the effects of BDNF neutralization or TrkB kinase interruption on cell apoptosis and invasion. The secretory BDNF was detected in supernatant of cultured HepG2 and HCCLM3 cells. BDNF content in HCCLM3 cells was more than that in HepG2 cells, which probably correlated with the high metastatic potential of HCCLM3 cells. Specific neutralizing antibody has been used in suppressing cytokine functions during variable biological processes [29]. We found in this study that cell apoptosis was significantly induced in anti-BDNF treated cells, which indicated that BDNF was required for supporting the survival of HepG2 and HCCLM3 cells. The involvement of BDNF in the invasion of HepG2 and HCCLM3 cells was also confirmed that invasive cells were evidently decreased by BDNF antibody. Studies have shown that inactivation of Trk by tyrosine kinase inhibitors was correlated with more apoptotic [30], or less invasive tumor cells [31], and aiming at interfering TrkB activation might be helpful in the development of effective anticancer therapies. K252a is a selective inhibitor of the tyrosine protein kinase activity of the trk family of oncogenes and neurotrophin receptors [32]. In this study, apoptotic cells were observed increasing after K252a treatment, which was considered that TrkB activated by BDNF was participated in the survival of HepG2 and HCCLM3 cells. Moreover, K252a used in this study also demonstrated a critical role of TrkB kinase activity in BDNF-induced invasion of HepG2 and HCCLM3 cells. Further investigations should be carried out for the detailed signalings downstream of BDNF/TrkB in regulating the survival and invasion of HCC cells.

Taken together, our study confirmed that both BDNF and TrkB were higher expressed in multiple HCCs, which was positively correlated with tumor progression. Secretory BDNF in supernatant of HCCLM3 cells with high metastatic potential were much more than that in HepG2 cells. Furthermore, HepG2 and HCCLM3 cells treated with BDNF neutralizing antibody or Trk tyrosine kinase inhibitor K252a showed increased apoptosis and decreased invasion. Our data thus revealed an important role of BDNF/TrkB in regulating survival and invasion of HCC cells and probably provided new insight into the inhibition of BDNF/TrkB signaling as a target of anti-HCC therapies. Nevertheless, the signaling pathway(s) downstream of BDNF/TrkB that involved in metastasis of HCC required further studies.

## Conclusions

Our data suggested that BDNF/TrkB supports the survival of HCC cells, and seems to serve as a critical mediator in the progression of intrahepatic dissemination of HCC cells, and prevention of BDNF/TrkB signaling could be an effective way in HCC therapy.

## Competing interests

The authors declare that they have no competing interests.

## Authors' contributions

Dw G initiated the research, carried out the experiments and wrote the manuscript, Xz H contributed to the paper translation, Xf J helped with the experimental design and gave funding support, Hb Z, Wy S and L Z gave experimental instructions, and J L gave critical review of the manuscript. All authors read and approved the final manuscript.

## Supplementary Material

Additional file 1 **Clinicopathological characteristics of 65 HCC patients in detail**. Distribution, differentiation, stage and lymph node metastasis were included, as well as BDNF score and TrkB expression by immunohistochemistry in HCC specimens, which were statistically analyzed in Table 1 and Table 2. Click here for file
