# Supplementary material for: More expressions of BDNF and TrkB in multiple hepatocellular carcinoma and anti-BDNF or K252a induced apoptosis, supressed invasion of HepG2 and HCCLM3 cells
Source: J Exp Clin Cancer Res. 2011 Oct 14;30(1):97. doi: 10.1186/1756-9966-30-97 (PMC3212909; doi:10.1186/1756-9966-30-97)
Supplement: Additional file 1 — Clinicopathological characteristics of 65 HCC patients in detail. Distribution, differentiation, stage and lymph node metastasis were included, as well as BDNF score and TrkB expression by immunohistochemistry in HCC specimens, which were statistically analyzed in Table 1 and Table 2. [file 1756-9966-30-97-S1.DOC]

**Supplemental Table - Clinicopathological characteristics of 65 HCC patients, including distribution, differentiation, stage and lymph node metastasis in detail, as well as BDNF and TrkB expressions by immunohistochemistry, which were statistically analyzed in Table 1 and Table 2.**

| Patients | Distribution | Differentiation | Stage | Lymph node metastasis | BDNF score | TrkB expression |
| --- | --- | --- | --- | --- | --- | --- |
| 1 | solitary | well | I | - | 0 | - |
| 2 | solitary | moderate | IIIb | - | 2 | - |
| 3 | solitary | well | IIIc | + | 4 | + |
| 4 | solitary | poor | IIIa | - | 1 | - |
| 5 | solitary | well | II | - | 4 | - |
| 6 | solitary | well | IIIa | - | 4 | + |
| 7 | solitary | well | IIIc | + | 4 | + |
| 8 | solitary | moderate | IIIc | + | 2 | - |
| 9 | solitary | poor | I | - | 0 | + |
| 10 | solitary | well | IIIb | - | 1 | - |
| 11 | solitary | well | IIIc | + | 4 | + |
| 12 | solitary | well | IIIa | - | 4 | + |
| 13 | solitary | moderate | I | - | 0 | - |
| 14 | solitary | poor | II | - | 1 | - |
| 15 | solitary | poor | I | - | 4 | + |
| 16 | solitary | well | IIIc | + | 4 | + |
| 17 | solitary | well | II | - | 1 | - |
| 18 | solitary | moderate | IIIc | + | 2 | - |
| 19 | solitary | moderate | IIIa | - | 0 | - |
| 20 | solitary | well | II | - | 4 | - |
| 21 | solitary | well | I | - | 1 | - |
| 22 | solitary | poor | IIIa | - | 1 | - |
| 23 | solitary | well | IIIc | + | 4 | + |
| 24 | solitary | moderate | I | - | 0 | - |
| 25 | solitary | poor | IIIa | - | 2 | + |
| 26 | multiple | moderate | IIIc | + | 4 | + |
| 27 | multiple | well | IIIb | - | 4 | + |
| 28 | multiple | well | IIIc | + | 4 | + |
| 29 | multiple | moderate | IIIa | - | 4 | - |
| 30 | multiple | well | IIIa | - | 4 | + |
| 31 | multiple | poor | IIIb | - | 2 | + |
| 32 | multiple | poor | IIIc | + | 4 | - |
| 33 | multiple | well | IIIc | + | 4 | + |
| 34 | multiple | well | II | - | 4 | + |
| 35 | multiple | well | IIIc | + | 4 | + |
| 36 | multiple | well | IIIb | - | 4 | + |
| 37 | multiple | moderate | IIIc | + | 2 | + |
| 38 | multiple | well | II | - | 4 | - |
| 39 | multiple | poor | IIIa | - | 4 | + |
| 40 | multiple | well | IIIc | + | 4 | + |
| 41 | multiple | moderate | II | - | 1 | - |
| 42 | multiple | well | IIIa | - | 4 | + |
| 43 | multiple | poor | IIIc | + | 4 | + |
| 44 | multiple | moderate | IIIa | - | 4 | + |
| 45 | multiple | well | II | - | 0 | - |
| 46 | multiple | poor | IIIc | + | 4 | - |
| 47 | multiple | poor | II | - | 4 | + |
| 48 | multiple | moderate | IIIa | - | 4 | + |
| 49 | multiple | well | IIIa | - | 4 | + |
| 50 | multiple | moderate | IIIc | + | 4 | - |
| 51 | multiple | poor | IIIc | + | 1 | - |
| 52 | multiple | well | IIIb | - | 4 | + |
| 53 | multiple | moderate | II | - | 2 | + |
| 54 | multiple | moderate | IIIc | + | 4 | - |
| 55 | multiple | well | IIIc | + | 4 | + |
| 56 | multiple | moderate | IIIa | - | 4 | + |
| 57 | multiple | well | II | - | 0 | - |
| 58 | multiple | poor | IIIa | - | 4 | + |
| 59 | multiple | moderate | IIIc | + | 4 | - |
| 60 | multiple | well | II | - | 1 | + |
| 61 | multiple | well | II | - | 4 | - |
| 62 | multiple | moderate | IIIb | - | 4 | - |
| 63 | multiple | poor | IIIc | + | 4 | - |
| 64 | multiple | poor | IIIa | - | 2 | + |
| 65 | multiple | moderate | IIIc | + | 4 | + |
